# Effect of Multiple Intraperitoneal Injections of Human Bone Marrow Mesenchymal Stem Cells on Cuprizone Model of Multiple Sclerosis

**DOI:** 10.29252/ibj.22.5.312

**Published:** 2018-09

**Authors:** Mohsen Marzban, Kazem Mousavizadeh, Masoomeh Bakhshayesh, Nasim Vousooghi, Gelareh Vakilzadeh, Anahita Torkaman-Boutorabi

**Affiliations:** 1 Department of Neuroscience, School of Advanced Technologies in Medicine, Tehran University of Medical Sciences, Tehran, Iran; 2 Cellular and Molecular Research Center and Department of Molecular Medicine, Faculty of Advanced Technologies in Medicine, Iran University of Medical Sciences, Tehran, Iran; 3 Research Center for Cognitive and Behavioral Sciences, Tehran University of Medical Sciences, Tehran, Iran

**Keywords:** Cuprizone, Mesenchymal stem cells, Mice, Multiple sclerosis

## Abstract

**Background:**

Bone marrow mesenchymal stem cells (BM-MSCs) elicit neuroprotective effects, and their repair ability has been investigated in different experimental models. We aimed to investigate the effect of multiple i.p. BM-MSCs injections in the cuprizone model of multiple sclerosis in mice.

**Methods:**

Adult male C57BL/6 mice (n = 40) were fed a regular diet or a diet containing cuprizone (0.2% w/w) for six weeks. Bone marrow samples were taken from patients with spinal cord injury. BM-MSCs (2 × 10^6^ in 1 milliliter medium) were administered intraperitoneally for two consecutive weeks at the end of the forth weeks of cuprizone administration. Animals (n = 12) were perfused with 10% paraformaldehyde at the end of sixth week. The brains were sectioned coronally in 6-8-μm thickness (-2.3 to 1.8 mm from bregma). The sections were stained by luxol fast blue-cresyl violet, and images were captured via a microscope. Demyelination ratio was estimated in corpus callosum in a blind manner. A quantitative real-time PCR was used to measure the myelin basic protein gene expression at sixth week.

**Results:**

Histologically, cuprizone induced demyelination in the corpus callosum. Demyelinated area was diminished in the corpus callosum of cell-administered group. Cuprizone could decrease myelin-binding protein mRNAs expression in corpus callosum, which was significantly recovered after BM-MSCs injections.

**Conclusion:**

Our data indicated a remyelination potency of multiple i.p. BM-MSCs in the cuprizone model of multiple sclerosis in mice.

## INTRODUCTION

Multiple sclerosis, a chronic inflammatory disease of the central nervous system (CNS) with demyelination and gliosis, can affect young individuals[1]. Remyelination is the innate recovery process of demyelination and may recover nervous tissue from axonal apoptosis and prevents age-long incapability. Therefore, intensification of remyelination may be an important factor for improvement of the disease. However, there is no effective treatment available.

Evidence has indicated the repairing effect of stem cell transplantation in demyelination[2]. It has also been shown that bone marrow mesenchymal stem cells (BM-MSCs) have special tendency to release protein for repairing and repressing immune cells[3]. These multipotent cells are able to differentiate into various cells, such as osteocytes, adipocytes, and chondrocytes[4]. BM-MSCs can also differentiate into neural-like and oligodendroglial-like cells[5].

BM-MSCs have been suggested to enhance the restoration of oligodendrocytes and therefore remyelination[6]. These cells can also be transplanted intravenously, intraperitoneally, intra-articularly, intracardially, intrahepatically, and intranasally. Intravenous (i.v.) injection is a repeatedly reported way of BM-MSCs transplantation for immunomodulation[7-11]. MSCs are injured by complement after their contact with serum[12]. Trapping of BM-MSCs in pulmonary blood vessels decreases the percentage of cells attaining the target and enhances the danger of pulmonary micro-thrombus[13]. Disability and fatality might be enhanced in case of pulmonary thrombosis. Moreover, the instant blood-mediated inflammatory reaction after the transplantation of BM-MSCs can be a reason for declined cell survival and elevated chance of thromboembolism[14].

Considering the aforementioned aspects, the i.v. transplantation of BM-MSCs can reduce their efficacy and their migration potency, decline the general cell survival and enhance danger of undesired influence. The i.p. transplantation is effectively a safe and a further efficacious way of BM-MSCs administration for disorders within the abdominal cavity. The i.p. injection of stem cell transplantation is easy to carry out and has the outlook of extensive choice in clinical cell therapy and has a high impact on patient management. The i.p. injection of BM-MSCs has been shown to be significantly more effective than i.v. administration for ameliorating clinical signs in a rodent model of inflammatory bowel disease[15]. The i.p. injections of BM-MSCs have also been reported for their regenerative potency and capability to transfer therapeutic genes[16]. A previous work has demonstrated that the intracerebral or i.v. injection of BM-MSCs increases myelination in the cuprizone model by amplifying myelination[17]. However, the migration of i.p. administered BM-MSCs has not been reported in the mice model of multiple sclerosis induced by cuprizone. In the present study, we aimed to investigate the influence of BM-MSCs in the cuprizone model of demyelination.

## MATERIALS AND METHODS

### Animal model

Male C57Bl/6 mice (n = 40), 7-8 weeks old, were purchased from Pasteur Institute of Iran (Tehran). The animals were subjected to a 12:12 h daylight/darkness with free access to food and water. The procedures were performed in accordance with institutional guidelines for animal care and use. The Research and Ethics Committee of the School of Advanced Technologies in Medicine, Tehran University of Medical Sciences approved the experimental protocol (project number 93-03-87-25963). The mice were fed with cuprizone (0.2% w/w; bis-cyclohexanoneoxaldi hydrazone, Sigma-Aldrich, St. Louis, MO, USA) in powdered rodent lab chow for a period of six consecutive weeks, as described previously[18]. An additional group of mice that received powdered lab chow without the addition of cuprizone was also used as a non-lesion control. Animals (n = 10 in each group) were divided into four groups as follows: Group 1, control; group 2, multiple sclerosis model; group 3, multiple sclerosis model + PBS (sham); group 4, multiple sclerosis model + BM-MSCs.

### In vitro culture and characterization of MSCs

Human MSCs were generated by conventional lab procedures: after obtaining an informed consent from the patients, the cells were harvested from the iliac crest during stem cell therapy for spinal cord injury patients. Bone marrow aspirated tissue was diluted with 4 volumes of PBS, filtered and centrifuged to density gradient with ficoll. The mononuclear cells were disported from the interface and washed in PBS, then suspended in a medium and put into 25-cm cell culture flasks. The medium contained alpha-minimum essential medium (α-MEM) supplemented with 10% (vol/vol) FBS, 100 U/ml penicillin, and 100 mg/ml streptomycin. The cells were cultured in 5% CO_2_, 85% humidity, at 37 °C. After 2 days of initial culture, non-adherent cells were removed, and adherent cells (80-90% confluence) were passaged. Medium replacement was done every 3-4 days. Grown colonies of adherent cells were separated with 0.025% Trypsin-EDTA and subcultured at a density of 2,000 to 5,000 cells/cm[19]. Cells were then passaged at 90% confluence and evaluated for cell surface phenotype beginning after the fourth passage.

### Characterization of BM-MSCs

Cells were analyzed for the expression of BM-MSCs markers after forth passage using flow cytometry The Cultivated cells were trypsinized and washed with PBS. At a density of 5 × 10^6^, the cells were incubated with 20 μl FITC-conjugated monoclonal antibody for CD73 or with phycoerythrin (PE)-conjugated monoclonal antibodies for CD34, CD45, CD90, CD105, CD13, and CD49e (1 h at room temperature). Isotype control antibodies were utilized for deletion of any non-specific reaction. FACS analysis was performed with a FACScalibur flow cytometer (Becton Dickinson, USA)[20].

### Adipogenesis

Cells were incubated in α-MEM containing 20% FCS, 100 U/ml penicillin, 100 mg/ml streptomycin, 12 mM L-glutamine, 5 mg/ml insulin (Sigma-Aldrich), 50 mM indomethacin (Sigma-Aldrich), 1 mM dexamethasone (Sigma-Aldrich), and 0.5 mM 3-isobutyl-1-methylxanthine for 2 weeks. Cells were then fixed with 10% formalin at room temperature for 20 min and stained with 0.5% Oil Red O (Sigma-Aldrich) in methanol (Sigma-Aldrich) at room temperature for 20 min [21].

### Osteogenic differentiation of MSCs

BM-MSCs were cultured in multi-well plates at 3 × 10^4^ cells per well in BM-MSCs medium. After 24 h, the medium was changed with osteogenic medium (BM-MSCs medium, 50 µM ascorbate-2-phosphate, 10 mM-glycerol phosphate, and 100 nM dexamethasone (termed osteogenic medium). After ten days, samples were stained with Alizarin Red S[22].

### Q-bands by fluorescence using quinacrine

In the 6^th^ passage of BM-MSCs, slides of prometaphase chromosomes were prepared with a hypotonic solution (0.05 M, HCL) at 37.5 °C for 15 min and fixed with methanol-acetic acid solution for 1 h. The cell slides were incubated in quinacrine mustard solution at room temperature for 10 minutes, rinsed with Sorensen’s buffer (pH 6.8) and mounted in the same buffer. The slides were studied via a fluorescence microscope[23].

### BM-MSCs transplantation

Animals were administered 2 × 10^6^ BM-MSCs in 500 µl of PBS intraperitoneally for two consecutive weeks at the end of the forth weeks of cuprizone administration. All experimental groups were kept on the cuprizone diet for an additional two weeks, following the i.p. administration of either PBS (sham) or BM-MSCs.

### Tissue processing for histopathological analyses

At the end of sixth week of cuprizone diet, animals (n = 12) were perfused with 10% paraformaldehyde for immunohistochemistry and histology assays. Coronal brain slices (8 µm) between -2.3 to 1.8 mm from bregma were prepared according to Paxinos and Franklin’s stereotaxic atlas[24]. Sections were stained with luxol fast blue (Sigma-Aldrich) and investigated by light microscopy. For luxol fast blue staining stereology (area estimation), a possible starting point for a pilot study might be 10 to 15 sections. The Cavalieri estimator was performed using a point grid. Area of remyelination was used as an indicator for tissue myelination. Briefly, digital images were captured at the same time for all samples using identical exposure times and compensation settings[25]. The efficiency of BM-MSCs for being located at corpus callosum was assayed by labeling BM-MSCs with the red fluorescent dye DiI (Sigma-Aldrich). Nuclear staining was done utilizing DAPI to determine cells being in corpus callosum[26]. For each image, the region of interest was the field of view at 40× magnification.

### Quantification of mRNA expression

Quantitative real-time PCR was performed to quantify the level of MBP gene expression in corpus callosum. RNA from tissues was prepared following the manufacturer’s specifications (total RNA RNeasy Mini Kit for tissue from Qiagen, Germany). cDNA was synthesized using the High-Capacity cDNA Reverse Transcription (TAKARA PrimeScript™ cDNA Synthesis Kit, Japan). Real-time quantitative PCR analysis was performed using StepOne™ Real-Time PCR System equipped with the **SYBR Green** (Applied Biosystems, USA). The double delta Ct analysis procedure was utilized to determine differences in the expression of MBP gene between animal groups. Survey in the mRNA expression quantity was computed after normalization to β-actin[27]. The primer sequences employed were: MBP forward: CCATCCAAGAAGACCCCACA, MBP reverse: CCCCTGTCACCGCTAAAGAA, mouse β-actin forward: CGCCACCAGTTCGCCATGGA, and mouse β-actin reverse: TACAGCCCGGGGAGCATCGT.

### Statistical analysis

All data were expressed as the mean ± SD. Statistical analysis included one-way ANOVA and Duncan post-hoc test for post hoc comparison if appropriate. *p* < 0.01 was considered as statistically significant.

## RESULTS

### Isolation, expansion, and characterization of BM-MSCs

**Fig. 1:**
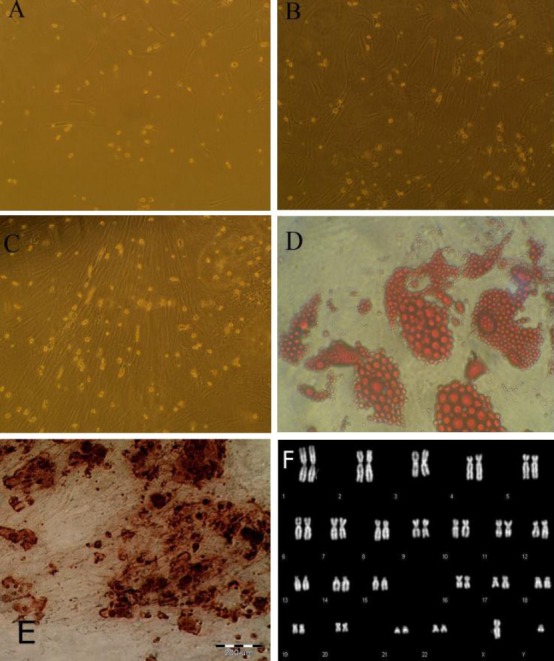
Stem cells from the bone marrow. Appearance and growth of fibroblastoid cells or bone marrow stromal stem cells at primary culture, passage 1 on days 3 (A), 7 (B), and 10 (C); adipose differentiation of BM-MSCs (D); osteogenic differentiation of BM-MSCs (E); Q-banding of human chromosomes (F).

Fibroblastic cells began to appear in the culture flasks five to seven days after plating bone marrow nucleated cells. The non-adherent hematopoietic cells in the culture were removed during the changes of medium. Initially, fibroblastic cells in a single colony were often separated from each other (Fig. 1A); however, after continuous culturing for one week, the number and the density of cells were greater in the colonies (Fig. 1B and 1C). In the sixth passage of BM-MSCs, a normal series of dark and brightly fluorescent regions of different sizes with human normal karyotype 46XY were seen (Fig. 1F).

### Adipose differentiation

After adipogenic induction, the cell morphology was changed from the elongated confluent fibroblastic cells to more oval shaped cells, which showed a distinct ring of red coarse vacuoles around the cell periphery after Oil Red O staining. These vacuoles appeared to be developed by day two and became more numerous and larger with time (Fig. 1D).

### Osteogenic differentiation of BM-MSCs

While growing in the osteogenic medium, BM-MSCs tend to aggregate and make “knotted formation”, which is visible by microscope. Mineralization of the aggregate is usually reported by compacted, refrangible sediment, which was assayed by measuring calcium deposition via Alizarin Red S staining (Fig. 1E).

### Flow cytometry analysis

**Fig. 2:**
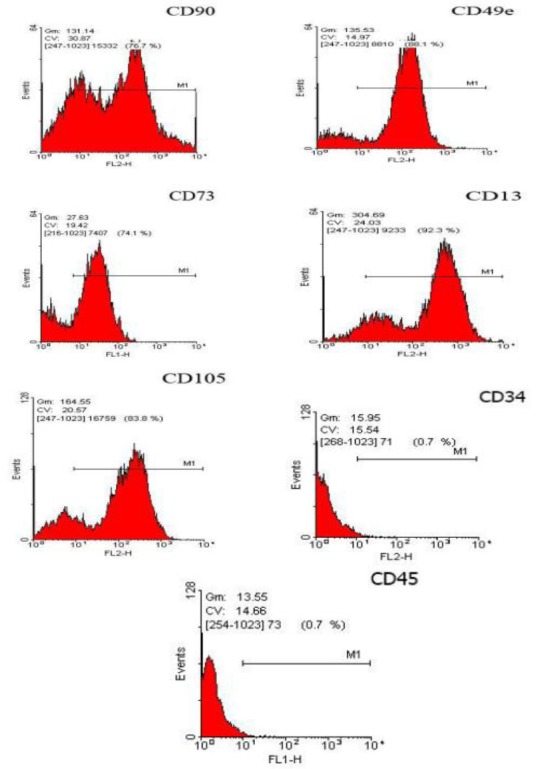
Flow cytometry histogram of the immunophenotype of BM-MSCs population. Expressions of five markers (CD90, CD49e, CD73, CD13, and CD105) and negative markers (CD34 and CD45) are shown.

The cells from passages four were tested by FACS analysis for the expression of the mesenchymal cells markers (CD73 CD90, CD105, CD13, and CD49e). More than 80% of the BM-MSCs derived from the bone marrow stem cell populations expressed the typical BM-MSCs marker proteins CD90, CD73, CD13, CD49e, and CD105. Also, more than 90% of the cells were negative for CD34 and CD45 (Fig. 2).

### Improving cuprizone-induced demyelination by BM-MSCs injection

**Fig. 3:**
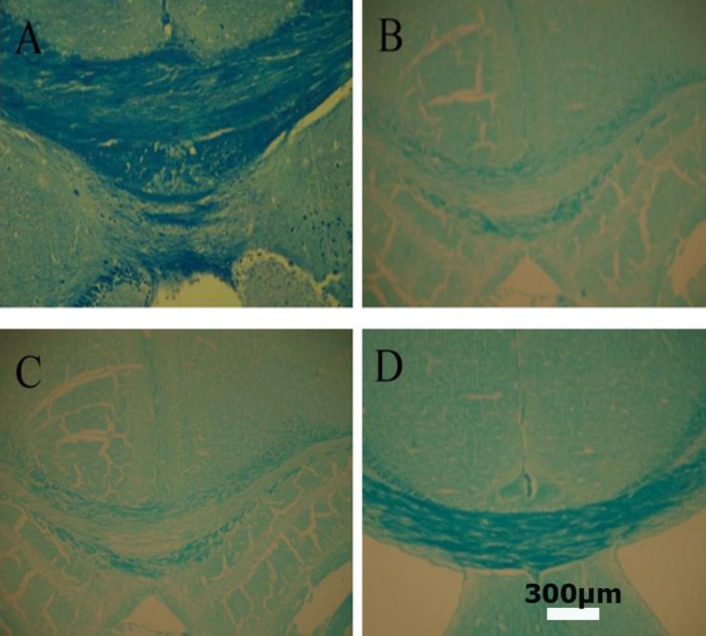
Histological examination of luxol fast blue myelin staining in corpus collasum. (A): Normal control; (B): cuprizone-induced demyelination (C): cuprizone-induced demyelination with PBS; (D): BM-MSCs induced remyelination. The scale bar is 300 μm

**Fig. 4:**
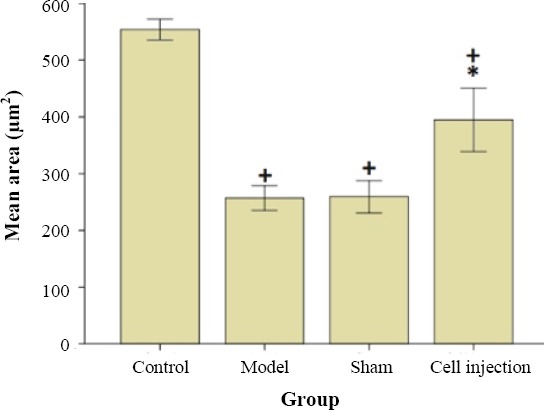
The effect of BM-MSCs i.p. administration on remyelination in cuprizone-induced demyelination. Each value represents mean ± SD. **^+^***p* < 0.01 compared to control group. *p < 0.01 compared to model and sham group.

Figures 3 and 4 show the effect of i.p. injection of BM-MSCs on cuprizone-induced demyelination. The remyelination was evaluated with the BioReport software and exhibited as quantitative form. Cuprizone-treated mice received either BM-MSCs (2 × 10^6^ cells/500 µl of PBS, i.p.) or an equivalent volume of PBS (sham) for two consecutive weeks, which was started at the end of the forth weeks of cuprizone administration. Staining of myelin with luxol fast blue displayed a steady and a profound loss of myelin within the corpus callosum of cuprizone exposed mice in the model and in the sham treatment groups, as compared to the stem cell-treated mice (Fig. 3). This analysis confirmed that cuprizone induced a significant loss of myelin in the corpus callosum (*p* < 0.01). BM-MSCs treatment provided a significant reduction in the demyelinating effects of cuprizone (*p* < 0.01) although demyelination was not completely remyelinated (*p* < 0.01). To investigate the probable mechanisms by which BM-MSCs restorate cuprizone-induced demyelination, we studied whether the i.p. administration of BM-MSCs migrates from the i.p. cavity to the brains of the injected animals. The DiI-labelled transplanted BM-MSCs were not observed in corpus callosum of the injected group.

### Analyses of RT-PCR data

**Fig. 5:**
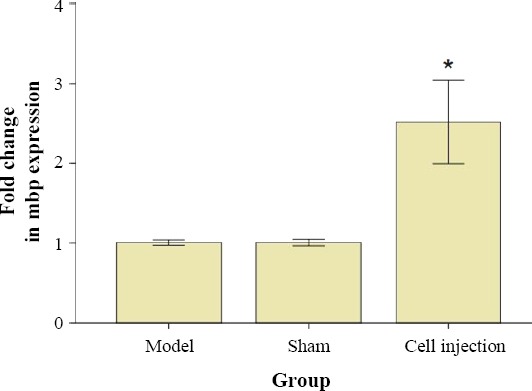
Expression level of MBP in the corpus collasom. MBP mRNA levels was studied by real-time PCR. Results were expressed as relative gene expression referred to the control and sham groups from data obtained using the equation: 2^-∆∆C^_T_. *p < 0.01 compared to the model and sham groups.

To explore the hypothesis that an alteration in MBP function is present in cuprizone-induced demyelination, we investigated the mRNA expression level of MBP using real-time PCR in the corpus callosum. The data analyses for the normalized expression of MBP gene are shown in Figure 5 and is expressed as the relative expression (fold) of increased or decreased of sham and transplanted groups compared to the control animals. In the BM-MSCs transplanted group, we found an increase in MBP mRNA level (*p* < 0.05) in comparison with the model and the sham groups. We did not observe any difference in the expression of house-keeping gene, β-actin, among the experimental groups. Three mice were used for each group. The qRT-PCR data were calculated as normalized relative quantity (RQ) values, with all RQ values for a gene being normalized to the mean RQ value of the respective model mice.

## DISCUSSION

Previous experiments using BM-MSCs in several models of multiple sclerosis led us to use the cuprizone model of multiple sclerosis. Since the cuprizone model is in drastic conflict to human multiple sclerosis, it does not mirror the complicated pathophysiology of the human multiple sclerosis where the peripheral immune system acts as a major player in injury propagation[28]. Nevertheless, animal models such as the cuprizone demyelination are effective in investigating the pathophysiology of remyelination, which is impossible in human. All rodent models only slightly imitate the pathophysiology of multiple sclerosis; therefore, different models have their advantages and disadvantages[29].

In the present study, we showed that the i.p.- administered BM-MSCs did not migrate to corpus callosum but improved myelination in this part of brain. However, another study showed that i.v. or i.p. injections result in rapid reduction in cell numbers. Few studies have also indicated the effect of mesenchymal stem cells on multiple sclerosis animal models[30]. Thus, administration of large amounts of cells or multiple injections might be required in these models. Nessler *et al*.[17] have demonstrated the efficacy of systemically injected BM-MSCs on cuprizone-induced demyelination in which the effects of intravenously and intranasally transplanted murine and human BM-MSCs were investigated but did not show any significant effect. They also suggested that BM-MSCs could not enter the CNS lesion in this model.

Our present results indicated that the i.p. administration of BM-MSCs improves myelination as it is shown in luxul fast blue staining and also increases MBP gene expression. In this study, we found that BM-MSCs did not migrate to injured site by DiI staining. However, these cells could significantly affect remyelination. Similarly, a previous study proved the potency of intracranially transplanted mesenchymal stromal cell to strengthen endogenous repair in chronic cuprizone-treated mice[31]. Oligodendrogenic and myelin restoration might be due to the soluble factors released by these cells[32]. In fact, more studies are demanded to determine the factors that are involved in the oligodendrogenic activity of BM-MSCs to enhance the regenerative property of these cells in multiple sclerosis. A number of studies in the experimental autoimmune encephalomyelitis (EAE) model have proved that stem cell transplantation could ameliorate EAE-inducing T-cell anergy[33,34]. Nevertheless, it is not obvious whether neuroprotection via BM-MSCs administration needs peripheral immune system for its influence. To investigate this, we utilized the toxic demyelinating cuprizone model. In contrast to EAE model for investigating demyelinating diseases, the cuprizone model may be employed for demyelination and remyelination, free of peripheral system effects[35]. Moreover, the peripheral immune system does not participate in cuprizone-induced demyelination process, which makes this model suitable for the present study[36].

We did not detect any BM-MSCs in rostral corpus callosum with i.p. administration. However, this route of administration elicited remyelination in the evaluated regions. Similarly, in EAE model, in which the peripheral immune system has a great role, stem cell administration suppressed the disease[37]. Therefore, we concluded that BM-MSCs may not have a direct effect on the CNS but may induce their effects by regulating the peripheral immune system. In this study, we used two experimental methods, histology analysis and quantitative real-time PCR, to test the effect of i.p. injection of BM-MSCs in cuprizone-treated mice. To study the level of MBP gene expression in the rostral region of the corpus callosum, we used an initial array screen of pooled RNA, followed by RT-PCR of transcripts[38].

MBP is an abundant component of the oligodendrocyte myelin membrane, alternatively spliced from the Golli-MBP gene. MBP is critical to myelin membrane biogenesis and to regulation of the entry of other proteins into the membrane sheets[39]. Our results indicated that i.p. administration of BM-MSCs had the potency of stimulating remyelination. BM-MSCs could not be found neither in the brain nor in the other place of CNS, indicating that human BM-MSCs do not migrate to CNS in cuprizone-treated mice. After systemic injection, the majority of BM-MSCs are eliminated through lungs and just a few stem cells pass to the peripheral blood stream[40]. It seems possible that the mechanism of modulation might be achieved by lungs. A recent experiment has proposed that effector and memory T cells are able to enter the lungs before migrating to the CNS[41]. This observation can indicate that BM-MSCs would communicate with effector T cells to affect their immunoregulatory ability. Moreover, in a previous experiment, BM-MSCs were directly transplanted into the CNS in the EAE model[36]. That study showed that BM-MSCs improves the disease symptoms and showed a similar result to intravenously transplanted cells in EAE, but they did not notice the disruption of BBB in that model which may result in free access of peripheral immune cells, growth factors, and cytokines to the brain and modulating the damage Thus, the direct influence of transplanted cells on CNS was not proved. Nevertheless, our data revealed that BM-MSCs had no direct effect. In contrast to our experiment, a recent research has shown that the i.v. administration of murine BM-MSCs migrates to the brain and could induced remyelination in cuorizone demyelination model. Nonetheless, the authors did not perfused animals brain before dissection, and it is not clear if the discovered cells were located in vessels or in the CNS[32]. In another experiment, authors showed an improving effect of intravenously injected adipose MSCs on remyelination. In this experiment, the corpus callosum was dissected, and cell suspension became ready for FACS analyses of glial cell-like oligodendrocyte progenitor cells. Moreover, in that study, animals were not perfused before the brain dissection, and blood cells were discovered in cell suspension[42]. In the present study, we showed that BM-MSCs do not perform any direct action on remyelination in CNS. We therefore can suggest that the peripheral immune system could be responsible for stimulating the effect of BM-MSCs on regenerative process in CNS inflammatory disease and injury. In addition, in i.p. transplantation strategies, BM-MSCs can release anti-apoptotic factors such as insulin-like growth factor, brain-derived neurotrophic factor, vascular endothelial growth factor, granulocyte-macrophage colony-stimulating factor, fibroblast growth factor-2, and transforming growth factor-beta in peritoneal cavity[43]. BM-MSCs can suppress the proliferation of T-cells and the release of inflammatory factor into blood circulation from peritoneal cavity. Moreover, BM-MSCs can induce remyelination in brain local micro-environment where they are able to enhance protection and repair[44].

We utilized multiple i.p. injections of BM-MSCs in the cuprizone model of multiple sclerosis and showed that this method can induce remyelination within two weeks. A reasonable therapeutic method of multiple sclerosis must consider all the pathophysiological processes of the disease and must be multipurpose. In establishing EAE model, BM-MSCs have demonstrated their potency in supplying an immunomodulatory action. Nevertheless, in other models of demyelination, which are not related to immune system, these cells can have beneficial ability to repair demyelination through immune-independent process. To the best of our knowledge, this is the first experiment demonstrating that multiple i.p. injection of BM-MSCs can ameliorate demyelination in the cuprizone model of multiple sclerosis through stimulating remyelination process.
